# P072 A case of Salmonella aortitis: a rare but important mimic of large vessel vasculitis that can be fatal if missed

**DOI:** 10.1093/rap/rkag113.102

**Published:** 2026-11-03

**Authors:** Tony Yap, Thushyanthan Guruparan, Erica Gurung, Mariam Malik

**Affiliations:** Basingstoke and North Hampshire Hospital, Basingstoke, United Kingdom; Basingstoke and North Hampshire Hospital, Basingstoke, United Kingdom; Basingstoke and North Hampshire Hospital, Basingstoke, United Kingdom; Basingstoke and North Hampshire Hospital, Basingstoke, United Kingdom

## Abstract

**Introduction:**

Giant cell arteritis (GCA) can affect cranial vessels and can also involve the aorta and proximal branches; described as large vessel vasculitis (LVV). LVV without polymyalgia symptoms or cranial involvement can present a diagnostic challenge.

Inflammatory appearances of the aorta and/or its proximal branches on imaging can have several aetiologies including large vessel vasculitis, IgG4-related disease, rheumatoid arthritis, spondyloarthritis, paraneoplastic phenomena and infective causes such as syphilitic and bacterial aortitis. We present a case of bacterial aortitis, highlighting the need for rheumatologists to carefully consider broad differentials in such patients.

**Case description:**

A 73-year-old male with a background of seropositive rheumatoid arthritis (RA) on methotrexate (MTX) and hydroxychloroquine (HCQ) was admitted with pre-syncope following persistent vomiting, fever, poor oral intake and productive cough for 3 weeks. He was an ex-smoker with past medical history of unprovoked bilateral pulmonary emboli and knee and hip replacements. He had no known infectious contact and last travelled 6 months ago within Europe. He denied any diarrhoea, abdominal pain, back pain, rashes, oral ulcers, ocular symptoms, night sweats or unintentional weight loss. He had a small left knee effusion with no clinical concerns for septic arthritis following an orthopaedic review; there were no peripheral stigmata of infective endocarditis.

CRP was elevated at 146 mg/L with a white cell count (WCC) of 10.6 x10^9^/L. MTX was suspended on admission for suspected infection and intravenous co-amoxiclav was started empirically. Subsequently, his blood cultures grew Salmonella enteritidis. This prompted a switch of antibiotics to intravenous ceftriaxone with microbiology team input resulting in improvement in inflammatory markers. CT scan of chest, abdomen and pelvis was done to look for deep-seated infections. This revealed periaortic fat stranding suggestive of inflammatory changes around the aorta that could represent aortitis. A subsequent whole-body PET CT showed intense multifocal FDG uptake in the wall of the upper abdominal aorta suggestive of mycotic aneurysms.

Other relevant investigations of note include a borderline positive connective tissue disease screen, normal complements, normal creatinine kinase, a very small IgM Lambda monoclonal band on serum immunofixation, negative urinary legionella and pneumococcal antigens as well as a negative HIV and syphilis screen. CT scan of head and transthoracic echocardiogram were unremarkable.

A 6-week course of intravenous ceftriaxone was advised by the microbiology team with repeat imaging suggested by the vascular team in 4-6 weeks to confirm resolution of bacterial aortitis.

**Discussion:**

We present a scenario where rheumatologists were consulted on whether this patient may have aortitis from a rheumatological cause. We highlight the importance of considering broad differentials for LVV; in particular, infection should be ruled out in immunosuppressed patients presenting with an acute illness. Blood cultures, FDG-PET and echocardiogram are important for diagnosis of infection as a cause for aortitis and to exclude infective endocarditis. A procalcitonin may also be helpful. Autoimmune screen, IgG4 levels, infection screen including HIV and syphilis can help exclude other causes.

Up to 25% of Salmonella bacteriaemia can lead to endovascular infections with a predilection for seeding in atheromatous plaques of large vessels. Salmonella aortitis is a rare condition described in the literature to account for around a third of infection-related aortitis with high mortality rates. Initial presentations described include constitutional symptoms, elevated inflammatory markers or pyrexia of unknown origin without gastrointestinal symptoms; this can make the diagnosis extremely challenging. Key risk factors including age, gender and smoking can increase the chances of widespread large-vessel atherosclerotic disease, providing the substrate for bacterial seeding. Delay in diagnosis and antibiotic initiation can significantly increase the risk of mortality from rapid aneurysm formation and rupture. Misdiagnosis for an autoimmune cause and inappropriate immunosuppression in such patients can be even more detrimental to the outcome. Isolation of the causative organism and prompt initiation of appropriate antimicrobial therapy are crucial for improving outcomes, with early involvement of the multidisciplinary team, including microbiology and vascular surgery specialists, as appropriate.

Management of Salmonella aortitis can depend on the severity and presence of complications; in our patient, early diagnosis in the absence of aortic dissection allowed a trial of long-term antibiotic therapy with close monitoring and repeat imaging. More severe cases with delay in diagnosis, extensive disease or complications may require surgical intervention.

**Key learning points:**

1. Large-vessel inflammation has a broad differential diagnosis, including autoimmune, paraneoplastic, and infectious causes. In patients presenting with non-specific symptoms and imaging evidence of large-vessel inflammation, these alternative aetiologies should be carefully considered before attributing the findings to large-vessel vasculitis secondary to giant cell arteritis.

2. Blood cultures, FDG-PET and echocardiogram are important for diagnosis of bacterial aortitis and to exclude infective endocarditis, an important differential here. Autoimmune screen, IgG4 levels, infection screen including HIV and syphilis can help exclude other causes.

3. Salmonella aortitis is often a diagnostic challenge and requires high index of suspicion. It should be considered in older patients with immunosuppression and/or cardiovascular risk factors presenting with constitutional symptoms, pyrexia of unknown origin or elevated inflammatory markers of unknown cause.

4. Early blood cultures allow prompt isolation of the organism as once it becomes contained deep within atheromatous plaques; subsequent cultures can become falsely negative.

5. Prompt diagnosis and timely treatment with appropriate antibiotics, along with involvement of the relevant teams like microbiology and vascular surgeons is crucial. This helps prevent complications and death from rapid aneurysm formation and rupture.

